# Occipital nerve block is effective in craniofacial neuralgias but not in idiopathic persistent facial pain

**DOI:** 10.1007/s10194-012-0417-x

**Published:** 2012-03-03

**Authors:** T. P. Jürgens, P. Müller, H. Seedorf, J. Regelsberger, A. May

**Affiliations:** 1 Department of Systems Neuroscience, University Medical Centre Hamburg-Eppendorf, Martinistrasse 52, 20246 Hamburg, Germany; 2 Department of Dental Prosthetics, University Medical Centre Hamburg-Eppendorf, Martinistrasse 52, 20246 Hamburg, Germany; 3 Department of Neurosurgery, University Medical Centre Hamburg-Eppendorf, Martinistrasse 52, 20246 Hamburg, Germany

**Keywords:** Trigeminal neuralgia, Facial pain, Trigeminal neuropathic pain, Occipital nerve block, Occipital, Neuralgia

## Abstract

Occipital nerve block (ONB) has been used in several primary headache syndromes with good results. Information on its effects in facial pain is sparse. In this chart review, the efficacy of ONB using lidocaine and dexamethasone was evaluated in 20 patients with craniofacial pain syndromes comprising 8 patients with trigeminal neuralgia, 6 with trigeminal neuropathic pain, 5 with persistent idiopathic facial pain and 1 with occipital neuralgia. Response was defined as an at least 50% reduction of original pain. Mean response rate was 55% with greatest efficacy in trigeminal (75%) and occipital neuralgia (100%) and less efficacy in trigeminal neuropathic pain (50%) and persistent idiopathic facial pain (20%). The effects lasted for an average of 27 days with sustained benefits for 69, 77 and 107 days in three patients. Side effects were reported in 50%, albeit transient and mild in nature. ONBs are effective in trigeminal pain involving the second and third branch and seem to be most effective in craniofacial neuralgias. They should be considered in facial pain before more invasive approaches, such as thermocoagulation or vascular decompression, are performed, given that side effects are mild and the procedure is minimally invasive.

## Introduction

The use of occipital nerve block (ONB) has been propagated in occipital and cervical pain syndromes and dates back into the 1970s [1]. Its use was later established in cervicogenic headache, where pain reduction following occipital nerve block is currently considered part of the diagnostic procedure [2–5]. Subsequently, ONBs were used in other primary headache like migraine [5–8], cluster headache [9–12] and chronic daily headache, respectively, tension-type headache [9, 13] (for review see [14]). The exact mechanisms by which ONB exerts its effects remain uncertain. In occipital neuralgia, local perineural application of anesthetics and steroids could cause a direct depolarisation and inhibition of neural excitability. However, this would not explain the effect in headaches involving the first trigeminal branch. One potential explanation is the concept of functional connectivity between high nociceptive afferents (C1-3) and trigeminal nociceptive afferents from the first branch with convergence in the trigemino-cervical complex (TCC) [15, 16]. The ONB is thought to interfere with the subsequent sensitization developing during the event of acute headache attacks, modulating the excitability of second-order neurons receiving input from both trigeminal and cervical afferents upon stimulation of either afferent input [15, 16]. Recent studies additionally suggest that trigeminal branches innervating the meninges can have extracranial collaterals which could be a direct target for therapeutic interventions such as ONB [17].

Despite the effective application in headaches involving the first trigeminal branch, little is known regarding efficacy of ONB in craniofacial neuralgias (apart from occipital neuralgia), trigeminal neuropathic pain and persistent idiopathic facial pain. More precisely, little is known whether ONB is effective in painful conditions involving the second and third maxillary branch. One report dating back to the 1960s reported complete pain relief in postherpetic neuralgia after alcohol blocks of the greater occipital nerve in all six patients [18]. Three of them had involvement not only of the frontal but also of the mandibular and maxillary branch of the trigeminal nerve. In another case report, one patient with neuropathic facial pain in the first and second trigeminal branch due to progressive facial hemiatrophy had sustained benefit from a single ONB with lidocaine and methylprednisolone for 4 months [19].

Consequently, patients attending our outpatient department were identified who had medically intractable facial pain and received uni- or bilateral occipital nerve blocks. We wanted to elucidate whether:

1. ONB effects were principally confined to the first trigeminal branch or would also extend to the second and third branch.
2. ONB would be clinically meaningful effective in craniofacial neuralgias, neuropathies and persistent idiopathic facial pain
3. Differences in efficacy were syndromal, i.e. more or less efficacious in neuralgias than in persistent facial pain.

## Patients and methods

### Study design

Medical records of patients with facial pain or cranial neuralgias who presented to the facial pain clinic of the University Medical Centre Hamburg-Eppendorf between December 2009 and July 2010 were reviewed. Only patients who received an ONB in these conditions with appropriate clinical documentation were included in this retrospective chart review. Further inclusion criteria were: diagnosis of craniofacial neuralgia, trigeminal neuropathic pain or persistent idiopathic facial pain according to the criteria given below, stable preventative medication during follow-up period and at least 18 years of age. Patients were treated with an ONB due to impairment by acute exacerbations of pain.

### Patients

All patients had been seen by headache specialists (AM, TPJ) who established a diagnosis of craniofacial neuralgia including classical trigeminal neuralgia (IHS 13.1.1), symptomatic trigeminal neuralgia (IHS 13.1.2) and occipital neuralgia (IHS 13.8) and persistent idiopathic facial pain (IHS 13.18.4) according to the current ICDH-II criteria [20]. In trigeminal neuralgia, intense and brief pain paroxysms occur in one or more divisions of the trigeminal nerve lasting from less than a second to 2 min. These stereotyped attacks are intense sharp or stabbing. Attacks can be precipitated by trigger factors such as washing, eating, drinking or talking. In addition, contact with trigger areas such as the nasolabial fold or the chin can provoke attacks, which can also occur spontaneously. Symptomatic trigeminal neuralgia is defined by the presence of a structural lesion other than a neurovascular compression. Occipital neuralgia is defined as a paroxysmal stabbing pain in the distribution of the greater, lesser and/or third occipital nerve with tenderness over the affected nerve. ONBs typically ease the pain. Persistent idiopathic facial pain is defined as deep and poorly localized facial pain which is present daily and for the entire or most of the day. It is limited to one side of the face and frequently starts around the nasolabial fold and the chin and is not associated to sensory loss or other physical abnormalities. Clinical diagnostics including radiography of the face and the jaws are unremarkable.

As no diagnostic criteria for trigeminal neuropathic pain are given in the ICHD-II, the criteria by Zakrzewska [21] had to be fulfilled. They define trigeminal neuropathic pain as a continuous dull pain with sharp exacerbations in the trigeminal area that may radiate beyond. It can be provoked by contact with areas of allodynia and by light touch. Subjective or objective sensory loss is typical and vasodilatation and swelling can occur.

Patients had been routinely contacted (by either OF or PM) every 3–7 days after ONB and pain intensity and relevant changes had been noted as part of the outpatient clinics internal standard protocol in the medical files. These outcomes were assessed during telephone contact verbally as indicated by the patient. This routine was implemented to ensure that patients without clinical effects or exacerbation after transient improvement were rapidly seen again to initiate alternative therapy. A total of 20 patients (7 males, 13 females, mean age 58.2 ± 20.4 years, range 21–89 years) were identified who had received a total of 25 ONBs with appropriate documentation. Detailed information on demographical data is given in Table 1. One patient had been lost to follow-up after 12 days (TNP4).

**Table 1:** Demographical data on patients who received occipital nerve blocks in facial pain

ID	Age (years)	Gender	Diagnosis	Branch	Side	Duration (years)	Concomitant disease	Current medication
TN1	72	F	TN	V2, V3	Right	10	Hypothyreoidism Bronchial asthma Chronic gastritis Chronic paranasal infection	Carbamazepine 400–600 mg XR, l-thyroxine 75 mcg, ranitidine 150 mg, budesonide + formeterol inhalator
TN2	67	M	TN (symptomatic)	V2	Right	8	Multiple sclerosis Intermittent tachycardia	–
TN3	70	M	TN (symptomatic)	V1, V2, V3	Right	10	Encephalomyelitis disseminata Arterial hypertension COPD	Carbamazepine 1,200 mg
TN4	84	F	TN	V2	Right	20	Percutaneous thermocoagulation of the trigeminal ganglion in 07/06 with remaining trigeminal hypoesthesia Arterial hypertension Hyperlipidaemia Thyroid dysfunction	Carbamazepine 400 mg, nitredipine 20 mg, tritamterene 50 mg, iodide 200 mcg, simvastatin 80 mg, losartane 50 mg, hydrochlorothiazide 12.5 mg, doxycycline 200 mg
TN5	48	F	TN (symptomatic)	V3	Left	11	Symptomatic trigeminal neuralgia (right side) in the past (complete remission) Multiple sclerosis (1992) Bile duct stenosis of suspected autoimmune origin	Oxcarbazepine 1,800 mg, azathioprine 75 mg, interferon beta-1 44 mcg, oral contraceptive, pantoprazole, preceding week 4 × 1,000 mg methylprednisolone i.v.
TN6	55	M	TN	V3	Right	1	History of alcohol addiction for 20 years, currently abstinent Lumbar and cervical disc herniation	Gabapentin 1,200 mg, carbamazepine 300 mg, baclofen 10 mg, disulfiram 250 mg
TN7	89	F	TN	V2, V3	Right	25	Microvascular decompression (1999)	Metoprololsuccinate 95 mg, l-thyroxine 150 mcg, candesartan 16 mg, hydrochlorothiazide 12.5 mg, cinnarizin e 20 mg, dimenhydrinate 40 mg
TN8	59	F	TN	V2	Right	5	Arterial hypertension Allergic reaction to phenytoin	Prednisolon 80 mg, enalapril 2.5 mg, atenolol 25 mg
TNP1	62	F	TNP	V2	Left	20	Spondylolysis due to scoliosis Hypothyreoidism	l-thyroxine 80 mcg
TNP2	29	M	TNP	V2	Right	7	Attention deficit hyperactivity disorder Chronic recurrent sinusitis	Methylphenidate 30-40 mg, gabapentin 900 mg, doxepine 25 mg
TNP3	43	F	TNP	V3	Right	8	Allergic asthma	Gabapentin 3,600 mg/d, salmeterol 50 mcg + fluticasone 500 mcg inhalator
TNP4	80	M	TNP	V2	Left	1	Total knee replacement	Metamizole PRN
TNP5_1-2^a^	32	F	TNP	V1, V2	Right	2	Reconstruction of cruciate ligament	Gabapentin 3,300 mg/d, indometacin 50–200 mg, topiramate 150 mg, mirtazapine 15 mg, pantoprazol 40 mg
TNP6	21	F	TNP	V2, V3	Right	3	Migraine without aura Hypothyreoidism Cholecystolithiasis	Duloxetine 120 mg, pregabaline 300 mg, metoprolol 100 mg
FP1_1-4^a^	77	M	FP	V1–V3	Right	15 months	Recurrent gastric ulcera Chronic low back pain Prostatic carcinoma Unilateral kidney resection due to tumor	Gabapentin 300 mg, amitriptyline 60 mg, omeprazol 40 mg
FP2	67	F	FP	V1–V3	Left	1	None	Lamotrigine 3 × 100 mg, amitriptyline 75 mg XR
FP3	77	M	FP	V2	Right and left	2	Perforated gastric ulcer	–
FP4_1-2^a^	55	F	FP	V2	Left	5	None	Carbamazepine 600 mg, amitriptyline 20 mg, paracetamol up to 1,500 mg
FP5	21	F	FP	V2	Left	1	Chronic knee and lower back pain Obesity	Diclofenac 75 mg PRN
ON1	55	F	ON	C2	Left	1 week	History of subarachnoidal hemorrhage Depression	Pregabaline 100 mg

*F* female, *M* male, *V1* first trigeminal branch, *V2* second trigeminal branch, *V3* third trigeminal branch, *FP* persistent idiopathic facial pain, *TN* trigeminal neuralgia, *TNP* trigeminal neuropathic pain, *ON* occipital neuralgia

^a^Patients received repetitive GON blocks. Baseline data are given only for the first visit

### Experimental design

As this was a chart review, no experimental design was predefined. Due to standard operating procedures and a standardized documentation in our clinic, patients were treated in a uniform manner. However, composition of the local anesthetic and steroid mixture varied in some patients and some received only unilateral blocks (see Tables 2, 3, 4, 5 for details).

**Table 2:** Results of ONB in trigeminal neuralgia (TN)

ID	Sides/mixture/volume	Pre-ONB					Post-ONB 3 days							
		Tenderness over GON	Duration of attack (s)	Intensity (VAS)	Susceptibility to triggers	Hypoesthesia after ONB	% of pre-ONB pain	Duration of attack	Intensity (VAS)	Susceptibility to triggers	Duration of improvement (days)	Response (≥50%)	Painful?	Side effects?
TN1	2× (50 mg L; 4 mg D/3.5 ml)	Ipsilateral (less pronounced contralateral)	60	5/10	5/10	n.a.	100	n.a.	5/10	10/10	0	**–**	**–**	**+**
TN2	1× (20 mg L; 4 mg D/2 ml)	Ipsilateral (unilateral block)	≤1	4/10	2/10	Right+ (unilateral block)	100	1–2 s	4/10	0/10	0	**–**	**–**	**–**
TN3	2× (30 mg L; 4 mg D/2.5 ml)	None	Up to 120	10/10	0/10	Right+, left +	0	n.a.	0/10	n.a.	6	**+**	**–**	**–**
TN4	2× (30 mg L; 4 mg D/2.5 ml)	Ipsilateral	Up to 60	10/10	0/10	Right−, left−	30	4 s	3/10	0/10	3	**+**	**+**	**–**
TN5	2× (30 mg L; 4 mg D/2.5 ml)	Ipsilateral	≤1	10/10	8/10	Right+, left +	5	≤1 s	1/10	0/10	69	**+**	–	–
TN6	2× (40 mg L(1%); 4 mgD/5 ml)	Ipsilateral	5	4/10	5/10	Right+, left +	50	1–2	2/10	9/10	77^a^	**+**	**+**	**+**
TN7	2× (30 mg L; 4 mg D/2.5 ml)	Ipsilateral contralateral	1	7/10	5/10	Right+, left+	40	1	2/10	5/10	3	**+**	–	–
TN8	2× (30 mg L; 4 mg D/2.5 ml)	Ipsilateral	30	8/10	10/10	Right+, left+	20^#^	1 s	3/10	8/10	5	**+**	–	**+**

Response was defined as an improvement of at least 50% compared to pre-ONB pain. Sides: 1 indicates unilateral ONB ipsilateral to the side of pain, 2 indicated bilateral ONB

*L* lidocaine, *D* dexamethasone, *n.a.* not available

^a^Follow-up was stopped at 77 days post-ONB

^#^First follow-up after 5 days

**Table 3:** Results of ONB in occipital neuralgia (ON)

ID	Sides/mixture/volume	Pre-ONB				Post-ONB 3 days						
		Tenderness over GON	Intensity (VAS)	Susceptibility to triggers	Hypästhesia after ONB	% of pre-ONB pain	Intensity (VAS)	Susceptibility to triggers	Duration of improvement (days)	Response (≥50%)	Painful?	Side effects?
ON1	2× (30 mg L; 4 mg D/2.5 ml)	Ipsilateral	10/10	10/10	Right−, left+ (ipsilateral)	0	0/10	0/10	107	+	–	–

Response was defined as an improvement of at least 50% compared to pre-ONB pain. Sides: 1 indicates unilateral ONB ipsilateral to the side of pain, 2 indicated bilateral ONB

*L* lidocaine, *D* dexamethasone

**Table 4:** Results of ONB in trigeminal neuropathic pain (TNP)

ID	Sides/mixture/volume	Pre-ONB				Post-ONB 3 days						
		Tenderness over GON	Intensity (VAS)	Susceptibility to triggers	Hypoesthesia after ONB	% of pre-ONB pain	Intensity (VAS)	Susceptibility to triggers	Duration of improvement (days)	Response (≥50%)	Painful?	Side effects?
TNP1	2× (40 mg L (1%); 4 mg D/5 ml)	Contralateral	3/10	0/10	Right+, left+	100	3/10	0/10	0	**–**	**–**	**+**
TNP2	2× (30 mg L; 4 mg D/2.5 ml)	Ipsilateral	5/10	1/10	Right−, left+	40/100^a, #^	6/10	0/10	0	**–**	**–**	**+**
TNP3	2× (40 mg L (1%); 4 mg D/5 ml)	Ipsilateral contralateral	10/10	9/10	Right+, left+	40/100^a^	8/10	10/10	0	**–**	**–**	**+**
TNP4	2× (40 mg L (1%); 4 mg D/5 ml)	None	8/10	8/10	Right+, left+	50	5/10	8/10	12^§^	**+**	**+**	**+**
TNP5_1	2× (30 mg L; 4 mg D/2.5 ml)	n.a.	10/10	10/10	Right+, left+	<5%	2/10	0/10	3	**+**	**–**	**+**
TNP5_2	2× (30 mg L; 4 mg D/2.5 ml)	Ipsilateral contralateral	9/10	10/10	Right+, left+	15	2/10	0/10	6	**+**	**–**	**–**
TNP6	2× (40 mg L (1%); 4 mg D/5 ml)	Ipsilateral	9/10	5/10	Right+, left+	40^#^	4/10	0/10	4	**+**	**–**	**+**

Response was defined as an improvement of at least 50% compared to pre-ONB pain. Sides: 1 indicates unilateral ONB ipsilateral to the side of pain, 2 indicated bilateral ONB

*L* lidocaine, *D* dexamethasone, *n.a.* not available

^a^As pain ratings were unchanged after ONB, improvement to 40% was considered implausible and response was consequently rated negative with 100% of pre-ONB pain

^#^First follow-up after 4 days

^§^Patient was lost to follow-up after day 12

**Table 5:** Results of occipital nerve block (ONB) in persistent idiopathic facial pain (FP)

ID	Sides/mixture/volume	Pre-ONB			Post-ONB 3 days					
		Tenderness over GON	Intensity (VAS)	Hypästhesia after ONB	% of pre-ONB pain	Intensity (VAS)	Duration of improvement (days)	Response (≥50%)	Painful?	Side effects?
FP1_1	2× (30 mg L; 4 mg D/2.5 ml)	Ipsilateral	6/10	Right−, left−	5	1/10	11	+	–	–
FP1_2	2× (30 mg L; 4 mg D/2.5 ml)	None	4/10	Right−, left−	40/100^a^	4/10	0	–	–	–
FP1_3	2× (30 mg L; 4 mg D/2.5 ml)	Ipsilateral	7,5/10	Right−, left−	0	0/10	7^§^	+	–	–
FP1_4	2× (50 mg L(1%); 4 mg D/6 ml)	None	6/10	Right−, left−	80^%^	5/10	0	–	–	–
FP2	1× (30 mg L; 4 mg D/2.5 ml)	Ipsilateral (unilateral block)	8/10	Left−	100^#^	5/10	0	–	–	+
FP3	2× (30 mg L; 4 mg D/2.5 ml)	Ipsilateral contralateral	3,5/10	Right−, left−	100	3,5/10	0	–	–	–
FP4_1	2× (30 mg L; 4 mg D/2.5 ml)	Ipsilateral contralateral	8/10	Right−, left−	100	8/10	0	–	+	–
FP4_2	2× (50 mg L(1%); 4 mg D/6 ml)	Ipsilateral contralateral	6/10	Right+, left+	60^%^	5/10	0	–	–	–
FP5	2× (50 mg L(1%); 4 mg D/6 ml)	Ipsilateral	7/10	Right−, left−	100	7/10	0	–	–	–

Response was defined as an improvement of at least 50% compared to pre-ONB pain. Sides: 1 indicates unilateral ONB ipsilateral to the side of pain, 2 indicated bilateral ONB

*L* lidocaine, *D* dexamethasone

^a^As pain ratings were unchanged after ONB, improvement to 40% was considered implausible and response was consequently rated negative with 100% of pre-ONB pain

^#^First follow-up after 4 days

^%^First follow-up after 6 days

^§^Despite ongoing benefit, duration of beneficial effects was limited to 7 days as medication was changed after day 7

A positive response to ONB was defined as at least 50% improvement of pain according to the patient’s global rating at the first telephone contact after ONB. If more than one ONB was performed, mean improvement for all blocks was calculated and the patient was classified as responder only if mean improvement was at least 50%. All but 2 patients were contacted 3–5 days thereafter (FP1_4 and FP 4_2 on day 6). Data had been collected immediately after the ONB and every 3–7 days thereafter. If the benefit had been less than 50% for more than 2 telephone visits, the subject had been asked to visit the outpatient department again on short notice or advised to take an alternative therapy. Apart from improvement measured in percent of baseline pain, pain intensity on a verbal rating scale (VRS) from 0 (no pain) to 10 (worst imaginable pain) and susceptibility to triggers on a scale from 0 (unsusceptible to triggers) to 10 (highly susceptible to triggers) had been noted. Before ONB, the presence of local tenderness over the greater occipital nerve had been tested bilaterally. After ONB, the patient had been screened for the presence of occipital hypoesthesia and asked whether the procedure was painful. At follow-up, the patients had been routinely asked about the degree of improvement, current pain intensity, susceptibility to triggers, and side effects as part of standard medical care. Additionally, they were asked about any change in medication. As patients were contacted on a regular basis with narrow intervals of 3–7 days, outcome measures were determined solely by personal contact and not by means of a diary.

### Occipital nerve block

All occipital nerve blocks were administered by TPJ to minimize variation. Injections were placed halfway on the nuchal line between the occipital protuberance and the mastoid process and above the occipital ridge [11, 13]. After the greater occipital nerve was located, local tenderness was evaluated bilaterally. All but 2 patients (FP2, TN2) had received bilateral nerve blocks. Unilateral blocks had been given on request of the patients (known cardiac arrhythmia in TN2, explicit wish by FP2). A mixture of a local anesthetic (Lidocaine-HCl 1 or 2%, B. Braun Melsungen, Melsungen, Germany) and dexamethasone as steroid (Fortecortin injekt 4 mg, Merck Pharma, Germany) had been injected after protruding a 21-G needle until periosteal contact was established and aspiration had been negative. After 15 min, the presence of occipital hypoesthesia had been tested.

### Statistical analysis

Patient data were entered into an Excel 2007 (Microsoft, Redmond, WA, USA) spreadsheet for descriptive statistics. For pairwise comparisons of metric data *t* tests (paired or unpaired, two-tailed,) were used, categorical data were analyzed using 2 × 2 or 2 × 3 tables for Fisher’s Exact test (SPSS 18.0) with *p* < 0.05 regarded significant. Graphs were plotted with SigmaPlot2000 (Systat Software, Inc. San Jose, CA, USA).

## Results

A total of 25 ONBs were given to 20 patients with craniofacial neuralgias, trigeminal neuropathic pain and persistent idiopathic facial pain. Among them, 8 patients suffered from trigeminal neuralgia (40%), 6 patients from trigeminal neuropathic pain (30%), 5 patients from persistent idiopathic facial pain (25%) and 1 patient from occipital neuralgia (5%). The male:female ratio was 1:1.9. The affected nerve branches are given in Table 1.

### Response rates

**Fig. 1:**
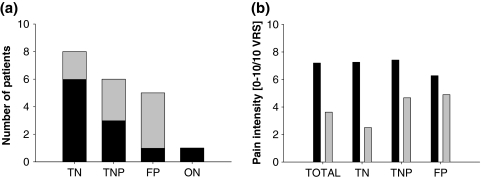
**a** Success rate of ONB among the different diagnoses. The height of *bars* indicates total number of patients; the *black parts* indicate patients with success. **b** Pain intensity ratings on a verbal rating scale (VRS) before and after ONB in various facial pain symptoms. *Black bars* indicate ratings before ONB, *gray bars* after ONB. *FP* persistent idiopathic facial pain, *TN* trigeminal neuralgia, *TNP* trigeminal neuropathic pain, *ON* occipital neuralgia

**Fig. 2:**
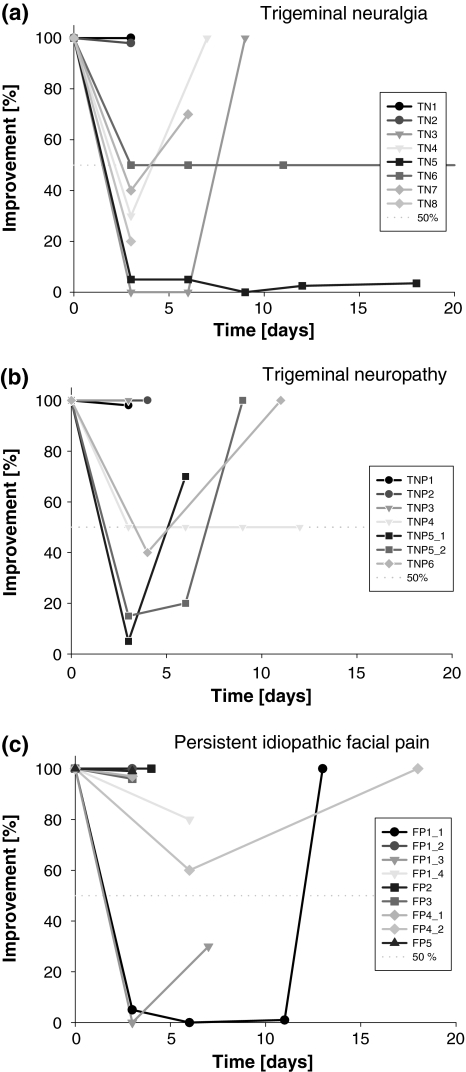
Individual response to ONBs indicated as remaining pain in percent of the initial pain before an ONB. **a** trigeminal neuralgia, **b** trigeminal neuropathic pain, **c** persistent idiopathic facial pain. In the following patients, response was set to values between 95 and 99% instead of the actual 100% for the sake of legibility: TN2, TNP1, FP3, FP4_1, FP5

A positive response with a global improvement of at least 50% pain from baseline according to the patient’s statement was found in 11 out of 20 patients (55%; Fig. 1). Response rates were highest in occipital neuralgia (1 patient with complete relief, i.e. 100%; Table 3) and trigeminal neuralgia with positive effects in 6 out of 8 patients (75%; Table 2; Fig. 2a). In trigeminal neuropathic pain, 3 out of 6 patients had a positive response into ONBs (50%; Table 4; Fig. 2b), in persistent idiopathic facial pain only 1 out of 5 patients responded to ONB (20%; Table 5; Fig. 2c). Two patients (TNP2 and FP1) reported improvement to 40% despite unchanged pain intensities. This constellation seemed implausible and consequently their response was rated as being negative. As for patients with multiple ONBs, TNP5 and FP1 were classified as responders, FP4 as non-responder.

Fisher’s Exact test using a 2 × 3 table with response (yes/no) versus type of facial pain (trigeminal neuralgia, trigeminal neuropathic pain, persistent idiopathic facial pain) yielded no significant result (*P* = 0.187).

Improvement of baseline pain 3 days after ONB compared between the different subtypes of facial pain yielded no significant results in pairwise comparisons (mean percent of baseline pain: trigeminal neuralgia 43.1 ± 38.8; trigeminal neuropathic pain 66.7 ± 38.8; persistent idiopathic facial pain: 85.3 ± 23.5). Results of unpaired *t* tests are as follows: trigeminal neuralgia versus trigeminal neuropathic pain: *p* = 0.283; trigeminal neuralgia versus persistent idiopathic facial pain: *p* = 0.053; trigeminal neuropathic pain versus persistent idiopathic facial pain: *p* = 0.375). Notably, the comparison between improvement in patients with trigeminal neuralgia and persistent idiopathic facial pain missed significance by a narrow margin.

Evaluation of the number of effective nerve blocks yielded similar results. The total response rate was 13 out 25 blocks (52%). In trigeminal neuralgia 6 out of 8 blocks (75%) were considered effective, in occipital neuralgia 1 out of 1 block (100%). In trigeminal neuropathic pain, 4 out of 7 blocks were beneficial (57%), in persistent idiopathic facial pain 2 out of 9 blocks were effective (22%).

Seven responders were females and 4 males (male:female = 1:1.8), which is comparable to the entire sample (1:1.9). Hypoesthesia ipsilateral to the pain was present in 10 successful ONBs and 3 unsuccessful ONBs (data on hypoesthesia were missing in TN1), no relevant hypoesthesia was noted in 4 successful and 7 unsuccessful ONBs (2 × 2 table with Fisher’s Exact test: *p* = 0.095).

### Pain ratings

Mean pain ratings for all patients (responders and non-responders) were reduced by 50% (VAS ratings pre-ONB 7.2, SD 2.4; post-ONB 3.6, SD 2.2; *t* test: *p* = 0.001; Fig. 1). The therapeutic effect was even higher in the subgroup of patients with trigeminal neuralgia with a reduction of 66% (pre-ONB 7.3, SD 2.7; post-ONB 2.5, SD 1.6; *t* test: *p* = 0.01). In trigeminal neuropathic pain, a reduction of only 37% was observed (pre-ONB 7.4, SD 2.8; post-ONB 4.7, SD 2.2; *t* test: *p* = 0.086). Reduction of pain intensity was lowest in patients with persistent idiopathic facial pain with only 22% (pre-ONB 6.3, SD 1.7; post-ONB 4.9, SD 1.9; *t* test: *p* = 0.140).

A comparison of net ONB effects on pain ratings (difference of pain ratings before and after ONB) between the subgroups yielded no significant results in unpaired *t* tests: trigeminal neuralgia versus trigeminal neuropathic pain *p* = 0.312; trigeminal neuralgia versus persistent idiopathic facial pain: *p* = 0.094; trigeminal neuropathic pain versus persistent idiopathic facial pain: *p* = 0.406).

### Sustained benefit

**Fig. 3:**
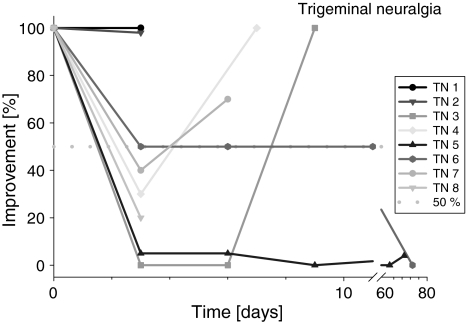
Individual response to ONBs indicated as remaining pain in percent of the initial pain before an ONB in trigeminal neuralgia including results of patients with sustained relief

Mean duration of clinical improvement (considering only patients with at least 50% improvement on day 3) was 27 days (range 3–107, SD 38.1). A sustained benefit (defined as continuous improvement of at least 50%) was obtained in 3 patients who were followed-up for 69, 77 and 107 days (Fig. 3). Thereafter, follow-up was suspended. It is noteworthy that all these patients suffered from craniofacial neuralgias (2 with trigeminal and 1 with occipital neuralgia). One of these patients (TN6) was able to successively reduce all preventative medication over the course of 77 days without recurrence of pain. As follow-up was eventually stopped in patients with long-lasting benefit (TNP4, TN6 and ON1), the number of days without improvement could even be higher (so far none of these patients has contacted our clinic for an appointment due to worsening of the symptoms, although we cannot exclude that the patient seeked further care at another specialized center).

### Tenderness over the greater occipital nerve

Neck pain was not reported by any patient. Local tenderness over the greater occipital nerve ipsilateral to the side of pain was present in all but three patients (two patients with trigeminal neuropathic pain, one with trigeminal neuralgia) and not tested in one patient (i.e. present in 83%). Contralateral tenderness was present in eight patients and not tested in one (i.e. present in 44%). A combination of the absent tenderness over the greater occipital nerve and missing hypoesthesia after ONB was found in only 2 unsuccessful blocks.

### Effect of repetitive ONBs

In three patients, repetitive ONBs were performed. However, only one patient (TNP5) reported recurrent benefit after both blocks, while another patient (FP1) experienced beneficial effects after two of four ONBs. In another patient (FP4), a second ONB was performed despite failure of the initial block again without success.

### Duration of attacks

In patients with trigeminal neuralgia, three patients reported shorter attacks, two patients an unchanged duration of attacks and another slightly longer attacks. Due to missing data, effects of ONB on attack duration could not be evaluated in 2 out of 8 patients.

### Susceptibility to triggers

The susceptibility to known triggers was lower in 3 out of 8 (38%) patients with trigeminal neuralgia and in 3 out of 6 patients with trigeminal neuropathic pain (50%) on the third day post-ONB. It was unchanged in two patients with trigeminal neuralgia and two with trigeminal neuropathic pain and increased in 2 patients with trigeminal neuralgia and 1 with trigeminal neuropathic pain. The only patient with occipital neuralgia reported a persistent and complete reduction of trigger factors after ONB with decline from 10/10 to 0/10. Trigger factors were not evaluated in patients with persistent idiopathic facial pain.

### Effect of unilateral blocks

Two patients who received unilateral ONBs did not respond.

### Effect of lidocaine dose

The amount of lidocaine and thus the applied volume varied over the observed period. Initially, 30 mg lidocaine was given routinely. Later, most patients received 40–50 mg. Distribution of response rates among various lidocaine amounts was not dose specific: a positive response was observed in 0 out of 1 ONB with 20 mg (0%), 10 out of 15 ONBs with 30 mg (67%), 3 out of 5 ONBs with 40 mg (60%) and 0 out of 4 ONBs with 50 mg (0%).

In addition, no relation between the lidocaine dose and the absence of hypoesthesia could be found. In 8 of 15 blocks (53%) with 30 mg lidocaine no hypoesthesia could be found and in 2 of 4 blocks (50%) with 50 mg and missing data in 1 patient. Hypoesthesia could always be found in ONBs with 20 mg (1 block) and 40 mg (5 blocks) lidocaine.

### Effect of affected trigeminal branches

Positive response rates were reported in the only patient with involvement of V_1+2_ (100%), in 2 out of 3 with involvement of V_1–3_ and V_2+3_ (67%), 3 out of 9 with V_2_ (33%) and 2 out of 3 with V_3_ (66%), respectively. The only patient with involvement of the greater occipital nerve (C_1–3_) responded (100%).

### Side effects

The injection procedure was rated painful by 2 out of 8 patients with trigeminal neuralgia (13%) and by one patient each with trigeminal neuropathic pain (17%) and persistent idiopathic facial pain (20%) resulting in a total of 4 out of 20 patients (20%). Transient and only mild ONB-related side effects were observed in 10 of the 20 patients (50%, see Table 6). Cranial flush or heat sensation was the most frequent side effects in 5 patients (25%), followed by a transient and mild bleeding after the syringe was pulled out in 2 patients (15%). All patients recovered completely without sequelae. One patient (FP4) suffered from dizziness and increased sweating on the third day after the first ONB. These were regarded as prodromal symptoms of a gastrointestinal infection, which was reported on the sixth day post-ONB and thus unrelated to ONB. Mean age of patients with side effects was 52 years (range 21–80 years, SD 19.8) and thus younger than the mean age of the entire cohort.

**Table 6:** Side effects of occipital nerve blocks graded according to their severity and outcome

Patient	Side effect	Severity	Outcome
Related to ONB			
TN1	Day 1: cranial heat sensation, mild local bleeding after removal of syringe	Mild	Complete remission
TN6	Day 0: mild transient hypertension	Mild	Complete remission
TN8	Day 0: cranial heat sensation	Mild	Complete remission
TNP1	Days 0–7: tenderness over injection side	Mild	Complete remission
TNP2	Day 1: cranial heat sensation, hypoesthesia left upper arm, day 4: tenderness over injection side	Mild	Complete remission
TNP3	Day 0: prolonged local bleeding for few minutes after syringe was pulled out	Mild	Complete remission
TNP4	Day 0: cranial heat sensation	Mild	Complete remission
TNP5_1	Day 0: dull cervical pain (right side)	Mild	Complete remission
TNP5_2	Day 0: bleeding after retrieving the syringe for 10 s	Mild	Complete remission
TNP6	Day 0: mild headache	Mild	Complete remission
FP2	Day 0: cranial flush, local tenderness over injection site (left occiput)	Mild	Complete remission
Unrelated to ONB			
FP4_1	Day 3: dizziness and increased sweating, most likely prodome of gastrointestinal infection reported on day 6	Mild	Complete remission

## Discussion

In this chart review, the effects of occipital nerve blocks were evaluated in 20 patients with craniofacial neuralgias or neuropathic pain and persistent idiopathic facial pain. All but one (occipital neuralgia) had second and/or third division trigeminal pain. The mean response rate was 55% (defined as at least 50% reduction of original pain reported by the patient) and was most effective in patients with trigeminal neuralgia (75%), although results did not reach statistical significance. ONB was less effective in trigeminal neuropathic pain (50%) and persistent idiopathic facial pain (20%).

Occipital nerve block reduced mean pain ratings significantly in the entire sample by 50% (again with greater effects in neuralgias and less pronounced effects in trigeminal neuropathic pain and persistent idiopathic facial pain). These effects lasted for an average of 27 days with a sustained benefit for up to 107 days in three patients (two with trigeminal neuralgia and one with occipital neuralgia).

Our data have to be seen with caution as our study was retrospective and open labeled. Groups were small and composition of the lidocaine/dexamethasone mixture varied. As such, these data mirror clinical routine and not a controlled study; consequently we decided to report descriptive statistics for most variables only. Despite these limitations, we show striking differences in efficacy of ONB between different entities of facial pain. Patients with idiopathic facial pain responded barely, while those with craniofacial neuralgia showed highest response rates.

### Pathophysiological implications

It has been shown in anatomical studies that A-delta and C-fiber afferents from trigeminal and occipital (C1-3) nerve branches terminate in the trigemino-cervical complex (TCC) [22–24] and that painful stimulation of either structure leads to up-regulation of metabolic activity and release of c-fos [24, 25]. Convergent neurons in the TCC have input not only from ipsi- but also from contralateral cervical afferents [16]. In our cohort, tenderness over the greater occipital nerve contralateral to the side of pain was present in 44% of all patients. As patients suffered from strictly unilateral and side-locked pain, this implies a clinically relevant bilateral connection. Based upon these findings, occipital nerve stimulation (ONS) is usually implanted bilaterally to avoid side changes observed in patients with cluster headache receiving ipsilateral ONS [26]. Therefore, bilateral nerve blocks could have higher efficacy and should be preferred if potential side effects do not preclude this option.

The phenomenon of referred pain in the occipital region upon nociceptive trigeminal activation has been described in patients with primary headaches such as migraine and cluster headache [27, 28]. The surprisingly high number of patients with tenderness at the greater occipital nerve ipsilateral to but well outside the painful area in our study is noteworthy. It suggests an important role of trigemino-cervical convergence of nociceptive afferents also in syndromes with involvement of the second and third (V_2_ and V_3_) trigeminal branch and could explain ONB efficacy in these conditions.

However, recent studies imply that ONB effects could be mediated by an additional mechanism. In rodents and human cadavers nociceptive collaterals of the nervus spinosus (meningeal branch of the mandicular nerve, formed by neurons from the maxillary and mandibular part of the Gasserian ganglion) were found to innervate both the dura mater and occipital periost and neck muscles [29]. Based on these observations, at least partial effects of ONBs in craniofacial pain could be conveyed by direct inhibition of extracranial collaterals of the maxillary and mandibular nerve. In addition, these results further corroborate the above-mentioned hypothesis that nociceptive V2 and V3 afferents converge with high cervical nociceptive afferents.

The observation that effects are most pronounced in craniofacial neuralgia and trigeminal neuropathic pain rather than in persistent idiopathic facial pain would argue in favor of different pathophysiological models. While craniofacial neuralgia [30] and trigeminal neuropathic pain [31, 32] are neuropathic pain syndromes with central components, no coherent construct exists in persistent idiopathic facial pain although one study reported neuropathic changes in patients with facial pain [31]. Interestingly, cortical somatosensory representation of the face was not altered in patients with persistent idiopathic facial pain and modalities of quantitative sensory testing were not affected [33]. It was therefore concluded that persistent idiopathic facial pain has elements of a central pain syndrome not sustained by somatosensory processing from the affected region.

### Technical considerations

#### Definition of response

Response was defined as an at least a 50% decrease in pain after ONB as judged by the patients in percent of baseline pain. Evaluation of interventional procedures based on subjective assessment by the patient is certainly controversial. Facial pain syndromes differ substantially in their temporal profiles (constant versus intermittent pain), pain characteristics (burning versus stabbing or lancinating pain) and other factors like reduced susceptibility to triggers. The above criterion had thus been chosen as standard in a clinical setting to increase comparability between groups and simplify evaluation for the patient. Moreover, reports of pain intensity yielded similar results.

#### Role of hypoesthesia

Occipital hypoesthesia after ONB was present in 76% of the patients. Hypoesthesia was absent in 8 out of 12 unsuccessful ONBs and present in 9 of 13 successful ONBs (which was statistically not significant). One potential explanation for missing hypoesthesia could be that ONBs were misplaced (as ONBs induce conduction blocks with consecutive sensory loss of the innervated area) or due to the varying amount of lidocaine/cortisone. However, there was no obvious correlation between the degree of hypoesthesia and the amount of lidocaine used.

Our data do at present not support a predictive role of hypoesthesia for ONB efficacy. This is in line with a previous study on cluster headache, migraine and new daily persistent headache [9] which found no significant association between occipital hypoesthesia and ONB efficacy.

#### Composition of anesthetic mixture

Lidocaine has been used frequently in ONB studies [2–6, 9, 10, 12]. Despite our positive results, we cannot exclude that results would have been better with bupivacaine [7, 34]. It not only has a significantly longer half-life than both prilocaine and lidocaine, but also a slower onset of action. However, it is doubtful if the half-life of the local anesthetic is crucial, as the clinical effects, when present, outlasted the local anesthetics’ half-life by far. Likewise, long acting steroids such as triamcinolon could be more advantageous in clinical practice.

Regarding the ideal composition of the injection it is not known whether a local anesthetic or corticosteroid alone or a combination of both is more effective. While some studies used either local anesthetics or corticosteroids alone (see [14] for review), the additional use of a steroid is important for ONB efficacy either by prolonging effects of local anesthetics or by acting independently on nerve activity [10, 13, 35]. The additional use of opioids and clonidine has been propagated but would need further studies to fully evaluate their therapeutic potential [3, 4].

Our limited data do not support dose-dependent effects of lidocaine within the limited range used in our study, which is in line with previous studies [36]. However, methylprednisolone was less effective in higher doses [37].

#### Injection site

Assuming that tenderness over the occipital region suggests vicinity to the greater occipital nerve, ONBs should have been placed correctly in our study in all but four patients. Although the greater occipital nerve is the main target in all published studies, the exact injection site varies substantially among the published studies. In some studies, ONBs were given below the occipital ridge [10], others have used higher and more lateral locations [9]. However, the anatomical course of the greater occipital nerve shows significant inter- and intra-individual differences [38]. In a human *post mortem* study, the exit point of the greater occipital nerve was located between 3 and 28 mm laterally to the occipital protuberance and 5–18 mm below the intermastoid line. Fixed injection schemes could be too static and may result in reduced efficacy of ONBs. For optimization, the use of nerve stimulators to locate the correct position is one option [2–4], ultrasound-guided techniques [39, 40] are another. For feasibility, we would suggest locating the exact position by tenderness on palpation (also referred to as TOP) which is a more practical approach (see [34] for review).

### Safety aspects

In our cohort, side effects were generally rare, mild, transient and completely remitting (see Table 6). No severe side effects occurred. Accidental intravenous injections of high dose local anesthetics have been reported to cause mild (such as lightheadedness or metallic taste) to severe (such as cardiac arrhythmia or epileptic seizures) adverse effects [41]. It should also be borne in mind that cutaneous atrophy has been reported in 1–14% of the patients receiving steroid injections [42]. Despite these rare side effects, ONB is a safe procedure in the hands of trained physicians.

### Limitations

Due to the design of our study as a retrospective chart review, several methodological shortcomings are inherent. As placebo effects observed in headache management can reach up to 50% in individuals [43] and invasive procedures are likely to have even higher placebo rates, we cannot exclude that our observations are mainly driven by placebo response. Nevertheless, sustained benefits in some patients lasting up to 2 months or more argue against pure placebo-mediated effects. Similar latencies have been observed in a small double-blind trial in patients with cervicogenic headache, who showed significantly prolonged pain-free periods after repeated occipital and supraorbital nerve block [3] and a single case with trigeminal neuropathic pain with substained benefit for 4 months [19]. Likewise, in a double-blind placebo-controlled trial a single suboccipital injection of betamethasone in patients with cluster headache led to prolonged effects with remission periods of up to 26 months [10]. Fluctuations in the natural course (as can be frequently seen in trigeminal neuralgia as an episodic disease) cannot be excluded and would require a controlled design. As only patients with complete datasets were included, this infers the risk of selection bias. In addition, results were not corrected for psychological comorbidity as this would be beyond the scope of a chart review.

### Implications for clinical practice and future perspectives

Treating patients with facial pain is challenging and may involve combining several drugs at higher dosages. Especially in elderly and frail patients preventive medication is often problematic as most of them are already on polypharmacy. In a sample of elderly internal patients in Austria, the mean number of drugs taken on admission was 7.5 per patient [44]. Adding more drugs can induce severe side effects and cause unpredictable interactions. Especially anticonvulsants (carbamazepin, oxcarbazepine, phenytoin, and valproate) or tricyclics (amitriptyline) can be hazardous in elderly as they induce or inhibit drug metabolism. Furthermore, most of these routinely used drugs cause side effects particularly problematic in elderly such as ataxia, arrhythmia and cognitive impairment. Thus, ONBs could be beneficial as a well-tolerated add-on therapy to bridge changes in preventive therapy and can lead to sustained effects alone in some individuals. However, it is important that results of this small retrospective trial should be interpreted with caution as subjects were not prospectively enrolled into a randomized placebo-controlled blinded trial.

## Conclusion

Occipital nerve block seems to be more effective in trigeminal neuralgia than in trigeminal neuropathic pain and persistent idiopathic facial pain. It seems plausible to use this method not only in patients with headache but also in patients with craniofacial neuralgias. Given that side effects are mild and that the procedure is minimally invasive, we suggest using this method before considering more invasive approaches such as thermocoagulation or vascular decompression. Moreover, it could be helpful for transient prophylactic treatment during dose escalation of first-line drugs (such as carbamazepine). However, as placebo effects are known to be high in chronic pain, results have to be interpreted with caution and randomized controlled studies are mandatory to confirm these preliminary results.
